# A High Incidence of Meiotic Silencing of Unsynapsed Chromatin Is Not Associated with Substantial Pachytene Loss in Heterozygous Male Mice Carrying Multiple Simple Robertsonian Translocations

**DOI:** 10.1371/journal.pgen.1000625

**Published:** 2009-08-28

**Authors:** Marcia Manterola, Jesús Page, Chiara Vasco, Soledad Berríos, María Teresa Parra, Alberto Viera, Julio S. Rufas, Maurizio Zuccotti, Silvia Garagna, Raúl Fernández-Donoso

**Affiliations:** 1 Programa de Genética Humana, Instituto de Ciencias Biomédicas, Facultad de Medicina, Universidad de Chile, Santiago, Chile; 2 Unidad de Biología Celular, Departamento de Biología, Universidad Autónoma de Madrid, Madrid, Spain; 3 Dipartimento di Biologia Animale, Università degli Studi di Pavia, Pavia, Italy; 4 Dipartimento di Medicina Sperimentale, Sezione di Istologia ed Embriologia, Università degli Studi di Parma, Parma, Italy; 5 Centro di Ricerca Interdipartimentale di Ingegneria Tissutale e Centro di Eccellenza in Biologia Applicata, Università degli Studi di Pavia, Pavia, Italy; Stowers Institute for Medical Research, United States of America

## Abstract

Meiosis is a complex type of cell division that involves homologous chromosome pairing, synapsis, recombination, and segregation. When any of these processes is altered, cellular checkpoints arrest meiosis progression and induce cell elimination. Meiotic impairment is particularly frequent in organisms bearing chromosomal translocations. When chromosomal translocations appear in heterozygosis, the chromosomes involved may not correctly complete synapsis, recombination, and/or segregation, thus promoting the activation of checkpoints that lead to the death of the meiocytes. In mammals and other organisms, the unsynapsed chromosomal regions are subject to a process called meiotic silencing of unsynapsed chromatin (MSUC). Different degrees of asynapsis could contribute to disturb the normal loading of MSUC proteins, interfering with autosome and sex chromosome gene expression and triggering a massive pachytene cell death. We report that in mice that are heterozygous for eight multiple simple Robertsonian translocations, most pachytene spermatocytes bear trivalents with unsynapsed regions that incorporate, in a stage-dependent manner, proteins involved in MSUC (e.g., γH2AX, ATR, ubiquitinated-H2A, SUMO-1, and XMR). These spermatocytes have a correct MSUC response and are not eliminated during pachytene and most of them proceed into diplotene. However, we found a high incidence of apoptotic spermatocytes at the metaphase stage. These results suggest that in Robertsonian heterozygous mice synapsis defects on most pachytene cells do not trigger a prophase-I checkpoint. Instead, meiotic impairment seems to mainly rely on the action of a checkpoint acting at the metaphase stage. We propose that a low stringency of the pachytene checkpoint could help to increase the chances that spermatocytes with synaptic defects will complete meiotic divisions and differentiate into viable gametes. This scenario, despite a reduction of fertility, allows the spreading of Robertsonian translocations, explaining the multitude of natural Robertsonian populations described in the mouse.

## Author Summary

Cells have different mechanisms to assess the proper occurrence of cellular events. These mechanisms are called checkpoints and are involved in the surveillance of processes such as DNA replication and cell division. A checkpoint at the pachytene stage arrests meiosis when defects in the process of homologous chromosome synapsis and recombination are detected. In mammals, both transcriptional inactivation of chromosomal regions that are not correctly synapsed at pachytene and activation of sex chromosome genes that are normally silent during this stage could contribute to meiotic arrest. We found that when Robertsonian translocations appear in heterozygosis, many synapsis defects occur, and mechanisms that trigger transcriptional silencing of the unsynapsed chromatin are activated. However, meiotic prophase-I progression is not greatly compromised. This questions the ability of the meiotic checkpoints to halt meiosis progression when synapsis is not completed, allowing cells with synapsis defects to reach the first meiotic division. The fertility reduction of Robertsonian heterozygous mice seems to be mainly caused by errors detected by the metaphase-I checkpoint, when most of the spermatocytes die, rather than by synapsis defects. In an evolutionary context, a permissive pachytene checkpoint could contribute to increasing the chances of Robertsonian translocations to spread into natural populations.

## Introduction

A series of complex processes takes place during the first meiotic division, including pairing, synapsis, recombination and segregation of homologous chromosomes. Defects in any of these processes can affect the normal progression of meiosis, causing severe fertility reduction or even sterility [1]–[3]. This is a consequence of the existence of surveillance mechanisms that monitor the accurate progression of meiotic events and promote the removal of defective cells. Two main checkpoints have been proposed to act during the first meiotic division: the pachytene checkpoint, responsible for ensuring the correct occurrence of recombination and synapsis [2],[4],[5], and the metaphase-I or spindle checkpoint, which controls the precise segregation of homologous chromosomes [6],[7].

Although the process that eliminates meiocytes in metaphase-I and II might be similar to that acting during mitosis [6],[7], a clear understanding of the mechanisms that trigger the pachytene checkpoint is still lacking. Given the interdependence between meiotic recombination and synapsis, it has been difficult to ascertain the existence of separate checkpoints for these processes in mammals. Thus, many recombination-defective mutants exhibit a delay in synapsis and/or synaptic aberrations, and meiosis is aborted during the zygotene-pachytene transition [8]–[10]. Likewise, most mutants defective for synaptonemal complex (SC) components abort meiosis at pachytene with unresolved recombination processes [11]–[15].

In addition to the accumulation of unresolved recombination intermediates, unsynapsed chromosomal regions undergo a process of transcriptional inactivation called meiotic silencing of unsynapsed chromatin (MSUC) [1], [16]–[18]. The mechanisms involved in transcriptional inactivation are particularly well characterized in mammalian male meiosis, in which sex chromosomes undergo a special case of MSUC called meiotic inactivation of sex chromosomes (MSCI) [19],[20]. This process is initiated with the accumulation of BRCA1 protein on the unsynapsed axial elements (AEs). BRCA1 is a protein involved in DNA damage repair that allows the recruitment of other factors such as ATR, promoting the phosphorylation of H2AX at serine 139 on the surrounding chromatin [21],[22]. The inactivation of sex chromosomes, which affects the unsynapsed regions of both the X and Y chromosomes, comprises an additional plethora of chromatin modifications that includes: 1) histone modification [18],[23],[24]; 2) incorporation of specific histone variants [25],[26]; 3) specific incorporation of non-histone proteins [27]–[30]; and 4) accumulation of XIST RNA [31] and other families of non-coding RNAs [32].

The initiation of MSUC seems to also operate by the action of BRCA1 and ATR [17]. Furthermore, it has been reported that many chromatin modifications detected during MSCI are also involved in the inactivation of unsynapsed autosomes. This is the case of H2AX phosphorylation [17], histone H2A ubiquitination [18], methylation of histone H3 and H4, incorporation of histone H3.3 [26] and Maelstrom protein [29]. However, the role of other chromatin modifications in MSUC remains to be demonstrated.

On these grounds, it has been proposed that MSUC may interfere with the expression of genes necessary for the completion of meiosis and this would contribute to arrest the meiotic progression of pachytene spermatocytes with synapsis defects [17]. More recently, it has been suggested that extensive asynapsis and MSUC could also interfere with MSCI [33]. Indeed, activation of some sex chromosome-linked genes that should remain inactive during meiosis has been claimed as one of the causes of meiotic failure in some mouse models [1],[17],[34],[35]. Mahadevaiah and co-workers [33] have proposed that MSCI initiation could be impeded by the sequestration of MSUC triggering proteins like BRCA1 and ATR on extensively unsynapsed autosomes, a circumstance that would preclude these proteins to relocate to the unsynapsed AEs of the sex chromosomes. MSCI abrogation has thus been proposed as the primary cause of spermatocyte death in mouse models that typically arrest meiosis at the zygotene-pachytene transition, including many recombination-defective mutants [33]. Sequestration of BRCA1 has been proposed to occur also in female meiosis [36]. However, in both cases cells seem to tolerate a certain degree of asynapsis, since both spermatocytes and oocytes with a reduced number of asynapsed chromosomes are able to progress through first meiotic prophase without interfering with of MSUC or MSCI processes [33],[36].

In the house mouse (*Mus musculus domesticus*), individuals that are heterozygous for Robertsonian (Rb) translocations (the fusion of two acrocentric chromosomes) show reduced fertility. This reduction is strongly correlated with impairment of spermatogenesis and loss of meiotic cells [37]–[45]. Depending on the number and complexity of Rb heterozygosity (i.e. formation of trivalents, chains or rings), meiocytes may be eliminated during prophase-I [41], [46]–[48] or during metaphase-I and II [39], [47]–[50].

The synaptic behaviour of trivalents in Rb heterozygotes has been extensively analyzed by means of electron microscopy in a wide range of mammalian species, including mouse and humans [42], [44], [45], [47], [51]–[54]. During meiosis, heterozygous mice display a high frequency of pairing abnormalities including: 1) delay in synapsis completion of trivalents; 2) existence of a variety of heterelogous synaptic situations, both within and between trivalents and between trivalents and the sex chromosomes; and 3) persistence of unsynapsed regions in the trivalents throughout pachytene. Furthermore, a reduction of the recombination frequency and a decrease of chiasma interference in these hybrids have been demonstrated [40], [55]–[58]. However, little is known about the chromatin modifications associated with these synaptic disturbances.

The aim of this study is to ascertain the extent of MSUC during meiosis in Rb heterozygous mice and to evaluate the consequences of this cellular response on the meiotic progression of spermatocytes. We used males generated by crossing individuals of a standard karyotype (2n = 40) with homozygous individuals bearing eight Rb translocations (2n = 24), collected from natural populations in Northern Italy. The resulting hybrids (2n = 32) bear eight trivalents that exhibit different degrees of asynapsis during meiosis. We have combined the analysis of synapsis and recombination progression during male meiosis with the localization of some proteins involved in MSUC, i.e., γH2AX, ATR, ubiquitinated H2A, SUMO-1 and XMR, the latter two having only been reported to act in MSCI. Our results describe the kinetics of MSUC in Rb heterozygotes and highlight the capacity of spermatocytes with synaptic defects to pass through pachytene and progress to the metaphase stage.

## Results

### Synapsis and recombination/repair progression is normal in bivalents but slightly delayed in trivalents

**Figure 1:**
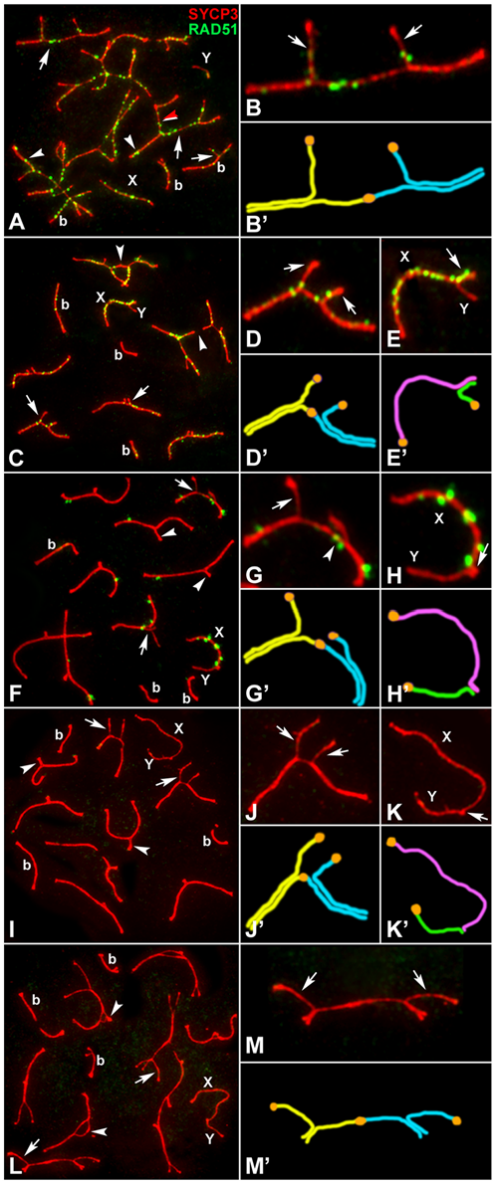
Localization of RAD51 (green) and SYCP3 (red) during prophase-I in 2n = 32 spermatocytes. (A) Zygotene. RAD51 is distributed in both synapsed LEs (arrowheads) and unsynapsed AEs (arrows) in bivalents (b) and trivalents. A distal heterologous association is observed between two trivalents (red arrowhead). Sex chromosomes (X, Y) appear separated. (B) Magnification of a trivalent shown in (A) with RAD51 foci on unsynapsed acrocentric (arrows) and metacentric chromosomes. Note that the synapsis starts only from one chromosomal end. (B') Schematic representation of the trivalent shown in (B). Homology between the metacentric and the acrocentrics is represented in yellow and blue and centromeres in orange. (C) Early Pachytene. Synapsis has been completed in bivalents (b). RAD51 focus starts to disappear from synapsed chromosomes but persist intensely in unsynapsed AE, X chromosome and trivalents. Most trivalents present an open configuration (arrows) and some of them show end-to-end connections (arrowheads). (D,D') Magnification of a trivalent shown in (C). RAD51 appears on synapsed and unsynapsed AEs (arrows). (E,E') Magnification of sex chromosomes shown in (C) and their schematic representation. RAD51 is located along the pseudoautosomal region (PAR) (arrow) and the unsynapsed AE of the X chromosome whereas it is absent from the Y chromosome AE. Synapsis occupies half of the Y chromosome. (F–H') Mid Pachytene. Trivalents show either open (arrows) or closed (arrowheads) configuration. RAD51 foci are scarce but still present, mostly on the synapsed regions of the trivalents and on the X chromosome. (G,G') Magnification of a trivalent with RAD51 in both synapsed and unsynapsed AEs. Notice that one of the open segments presents RAD51 (arrowhead) while the other does not (arrow). (H,H') Sex chromosomes: arrow marks the PAR. Synapsis is reduced if compared to that at early pachytene. (I–K') Late pachytene. RAD51 is completely absent. Again, trivalents can show either open configuration (arrows) or closed (arrowheads) configuration. The region of synapsis between sex chromosomes is reduced to a very distal short segment (arrow in K). (L–M') Diplotene. RAD51 is absent. Some trivalents rapidly start desynapsis (arrows) whereas others show heterologous distal association of the acrocentrics (arrowheads).

To characterize the progression of the first meiotic prophase, we used three main criteria: 1) the localization of SYCP3, the main component of the synaptonemal complex (SC) axial/lateral element (AE/LE), and that of RAD51, a protein related to early meiotic recombination and repair (Figure 1) that is abundantly incorporated along the chromosomes at zygotene, and then gradually disappears during pachytene and is absent at mid/late pachytene [59]; 2) the length of the pairing region between X and Y chromosomes, which extends up to 100% of the Y chromosome at early pachytene and becomes shorter as pachytene proceeds [60]; and 3) the reduction of the pairing region of sex chromosomes to the very distal end, the appearance of excrescences on the AEs of sex chromosomes, and the widening of SC attachment plates on the autosomes that identify the late pachytene stage. These criteria are comparable with those reported in recent studies carried out using RPA and MLH1 as markers of pachytene progression [61].

We found that synapsis was initiated at early zygotene in both bivalents and trivalents, but proceeded more quickly in the bivalents (Figure 1A). In fact, most trivalents were still undergoing synapsis when bivalents (b in Figure 1) were almost completely synapsed. This may be due to the fact that in trivalents, synapsis was initiated only at the distal ends of the chromosomes (Figure 1B–1B'). At this meiotic stage, the X and Y chromosomes usually lay apart from each other. At early pachytene, all bivalents and some trivalents had completed synapsis (closed configuration), although in many trivalents the chromosomal regions close to the centromeres were still unsynapsed (open configuration) (Figure 1C–1E' and Table 1).

**Table 1:** Number and frequency of cells showing open trivalents during pachytene and diplotene in Robertsonian heterozygotes.

Number of open trivalents	Early Pachytene		Middle Pachytene		Late Pachytene		Early Diplotene		Middle/late Diplotene	
	n	%	n	%	n	%	n	%	n	%
0	3	2.22	36	9.94	38	12.45	35	17.49	38	18.36
1	9	6.66	111	30.67	136	44.59	100	50.00	106	51.20
2	16	11.85	115	31.76	98	32.13	52	26.00	53	25.60
3	26	19.25	70	19.34	31	10.16	11	5.50	10	4.83
4	37	27.41	25	6.90	2	0.66	2	1.00	0	0
5	18	13.33	5	1.38	0	0	0	0	0	0
6	17	12.59	0	0	0	0	0	0	0	0
7	8	5.92	0	0	0	0	0	0	0	0
8	1	0.74	0	0	0	0	0	0	0	0
**Total**	**135**		**362**		**305**		**200**		**207**	

The pattern of RAD51 localization at the early stages of prophase-I was similar to that exhibited by mice with the standard acrocentric karyotype. During zygotene, a large number of RAD51 foci appeared on both synapsed and unsynapsed AEs of bivalents and trivalents and on the X chromosome (Figure 1A–1B'). Then, during early pachytene, the number of foci started to drop, although foci remained more abundant in both trivalents and sex chromosomes than in bivalents (Figure 1C–1E'). RAD51 foci were associated with the trivalents in either the open or closed configuration and did not preferentially accumulate on the unsynapsed regions of the open trivalents (Figure 1C–1D').

At mid-pachytene, many trivalents had completed synapsis and appeared in a closed configuration, but one to four trivalents remained in an open configuration (Figure 1F–1H'). At this stage RAD51 was only present on the sex chromosomes and on some trivalents.

At late pachytene, most cells exhibited up to four trivalents with an open configuration (Figure 1I–1K'). At diplotene, when desynapsis starts and homologues initiate their separation, the proximal ends of the acrocentric chromosomes remained associated in some trivalents while they appeared clearly separated in others (Figure 1L–1M'). RAD51 was not detectable at late pachytene (Figure 1I–1K') or diplotene (Figure 1L–1M'). These results indicate that the repair of DNA might be delayed in some trivalents as it is in the sex chromosomes, but this process seems to culminate successfully in mid-late pachytene, when the signal of RAD51 disappeared, even though many unsynapsed chromosome regions are present.

### Presence of heterologous synapsis

**Figure 2:**
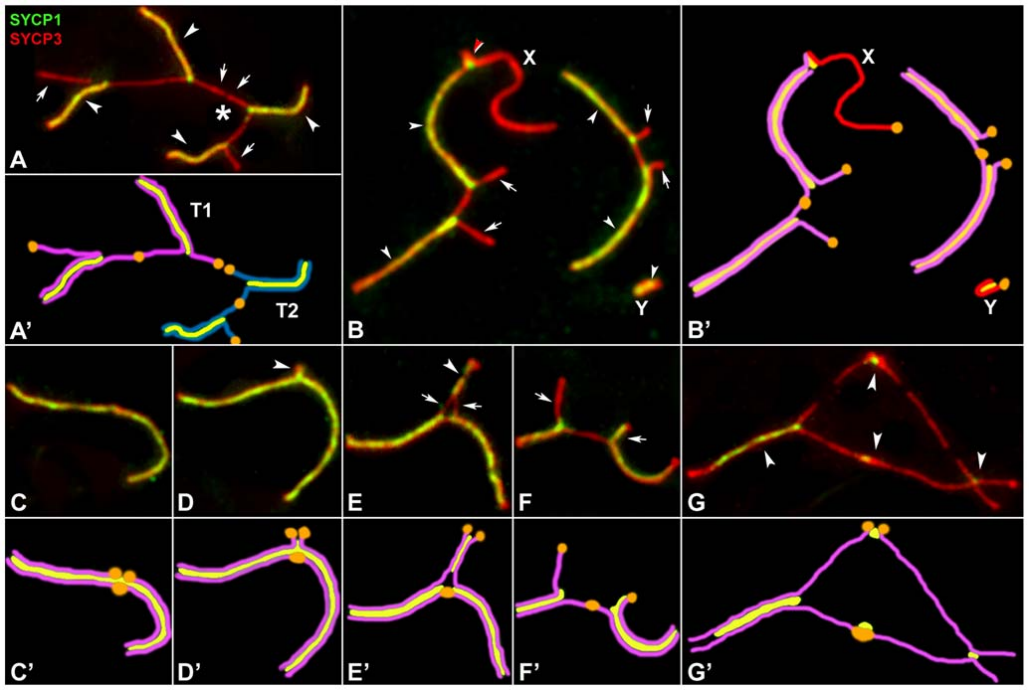
Synaptic conditions of Robertsonian trivalents during prophase-I. SYCP3 (red) and SYCP1 (green). (A,A') Zygotene trivalents (T1 and T2) and their schematic representation. The distal ends of the acrocentric chromosomes are synapsed with the metacentric chromosomes (arrowheads) whereas the proximal ends are unsynapsed (arrows). Two unsynapsed proximal ends are engaged in a heterologous end-to-end connection (asterisk). (B,B') Early pachytene. Trivalents show co-localization of SYCP3 and SYCP1 in the synapsed regions (arrowheads) whereas unsynapsed regions (arrows) that do not present SYCP1. One trivalent is associated with the X chromosome by a short segment of SC (red arrowhead). The single Y chromosome appears self-synapsed. (C,C') Complete homologous synapsis of two acrocentric chromosomes with the metacentric chromosome. (D,D') Heterologous synapsis between two acrocentric chromosomes involving a small chromosome region (arrowhead). (E,E') Partial heterologous synapsis of the acrocentric chromosomes involving a long chromosome region (arrowhead). Notice the unsynapsed chromosomal segment in the heterologous region (arrows). (F,F') Open trivalent. The proximal region of one acrocentric and the interstitial region of the metacentric chromosome do not present SYCP1, while the other acrocentric chromosome presents SYCP1 in its unsynapsed segment. (G,G') During diplotene, the heterologous synapsis between acrocentric chromosomes persists (arrowheads) even when desynapsis begins in the trivalent.

**Figure 3:**
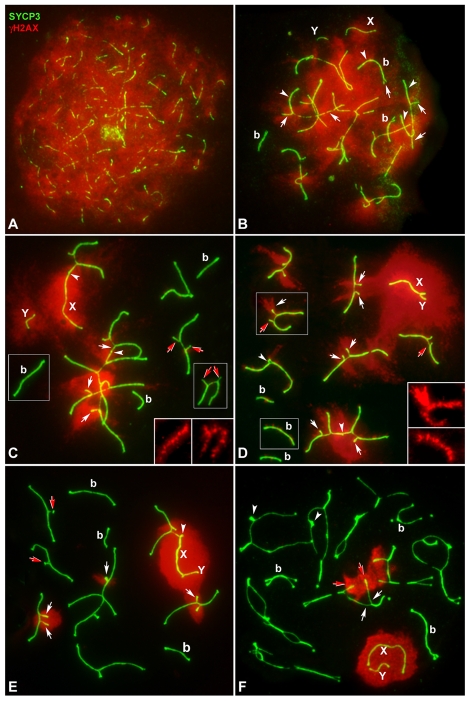
Localization of γH2AX (red) and SYCP3 (green) during prophase-I. γH2AX signal has been under-exposed to show its localization in unsynapsed regions. Inserts represent γH2AX labeling of selected bivalents and trivalents at a normal bright level. (A) Leptotene. γH2AX is distributed throughout the whole chromatin. (B) Zygotene. γH2AX is present in the X chromosome (X) and regions that are still unsynapsed (arrows), but fades in synapsed regions (arrowhead) of bivalents (b) and trivalents and is undetectable in the Y chromosome (Y). (C,D) Early Pachytene. γH2AX occupies a wide area in the chromatin surrounding the unsynapsed AEs of X, Y and autosomes (white arrows), but also localizes in the chromatin close to the SC in synapsed regions in both bivalents and trivalents. Some unsynapsed regions of trivalents do not present γH2AX signal (red arrows). Arrowheads marks ectopic synapsis between trivalents and between a trivalent and the X chromosome. Sex chromosomes lie apart from each other in (C) but have synapsed in (D). Notice that in the selected trivalent shown in (D) both acrocentrics are unsynapsed near the proximal region, but one of them shows an intense γH2AX labeling (white arrow) while the other lacks labeling (red arrow) (see insert). (E) Late pachytene. γH2AX is restricted to the chromatin surrounding the unsynapsed AEs of XY chromosomes and open trivalents (white arrows). Ectopic association is found between one acrocentric chromosome and the X chromosome (arrowhead). Red arrows mark the absence of γH2AX in synapsed heterologous regions of closed trivalents. (F) Diplotene. γH2AX labeling is present in two trivalents and the sex chromosomes. Labeled trivalents show two different regions: The chromatin proximal to the centromeres is labeled in both the acrocentric and the metacentric chromosomes (red arrows) while more distal regions are unlabelled (white arrows). In unlabelled trivalents, the proximal regions of acrocentrics often appear associated (arrowheads).

Trivalents commonly engage in ectopic heterologous associations with other trivalents and/or the sex chromosomes (Figure 1C). Thus, we wondered whether these associations would involve the assembly of the SC as a tripartite structure. For this purpose we analyzed the localization of SYCP1 protein, one of the main components of the SC transverse filaments and central element (Figure 2). At zygotene, we found that trivalents could establish an end-to-end connection that did not usually involve SYCP1 (Figure 2A–2A'). However, the association of unsynapsed proximal ends of trivalents with the sex chromosomes frequently involved the formation of a short SC with either the distal region of the X chromosome, the proximal region or both (Figure 2B–2B'; see also Figure 3 and Figure S1). Furthermore, the Y chromosome was sometimes found in a self-synapsed configuration (Figure 2B–2B' and Figure S1). These situations usually occurred at early pachytene and were more rarely detectable from mid-pachytene onwards.

Heterologous synapsis was also found within each trivalent. Although it was expected that the two acrocentrics could synapse with the corresponding homologous segment of the metacentric (Figure 2C–2C'), synapsis between the heterologous proximal chromosomal regions of the acrocentrics was the most frequent configuration. Heterologous synapsis could involve either a short (Figure 2D–2D') or a long segment of both chromosomes (Figure 2E-2E') and could be maintained from pachytene until late diplotene (Figure 2G–2G'). Furthermore, we found that some unsynapsed chromosomal regions incorporated SYCP1 (Figure 2F–2F'), perhaps representing either unsynapsed regions that were about to synapse or regions of self-synapsis. Alternatively, they may reveal only a non-specific binding of SYCP1 to unsynapsed AEs, a feature that is frequently observed in the sex chromosomes [62].

### Association of MSUC markers with unsynapsed trivalents

To evaluate the incorporation of MSUC markers on unsynapsed Rb trivalents, we first examined the temporal localization of γH2AX (Figure 3 and Figure S1; Video S1). This protein localizes in foci at DNA double-strand breaks during DNA repair and it is also associated with the inactivation of unsynapsed chromatin in autosomes and sex chromosomes [17],[63],[64]. At leptotene, the localization of γH2AX was dispersed throughout the nucleus (Figure 3A; Video S1); then, at zygotene, γH2AX began to disappear from the synapsed chromosome regions of the bivalents and from the synapsed distal regions of some trivalents (Figure 3B). The X chromosome appears intensely labeled while the Y chromosome is usually devoid of γH2AX labeling.

When spermatocytes entered pachytene, γH2AX became restricted to the chromatin located close to the LEs of the synapsed regions of both bivalents and trivalents (see insets in Figure 3C and 3D) [65]. In the sex chromosomes, γH2AX was extended over the chromatin (Figure 3C and 3D). Interestingly, we observed that the Y chromosome is intensely labeled even when it occasionally appears self-synapsed (Figure S1). In the unsynapsed regions of the trivalents, γH2AX showed two labeling patterns: 1) occupying a wide chromatin area surrounding the unsynapsed segments, as in the sex chromosomes, and 2) occupying a more restricted chromatin area, very close to the unsynapsed AEs, as in the synapsed regions (see inset in Figure 3C). It is especially striking that in some trivalents one of the unsynapsed acrocentric chromosomes showed one of these labeling patterns while the other acrocentric showed the alternative pattern (see inset in Figure 3D).

From mid to late pachytene, γH2AX labeling appeared as a bright signal on the entire chromatin surrounding the X and Y chromosomes and the AEs of the unsynapsed regions of open trivalents (Figure 3E). It is important to stress that open trivalents showed γH2AX labeling regardless of whether they were close to the X and Y chromosomes or far from them, indicating that this labeling was not a consequence of their association with the sex chromosomes (see Video S2). At diplotene the localization of γH2AX in the sex chromosomes remained visible, and it was also detectable in the pericentromeric regions of some trivalents (Figure 3F). These regions most likely represent chromosomal segments that have remained unsynapsed during pachytene, since those that began desynapsis during diplotene, in either bivalents or trivalents, were devoid of γH2AX labeling. These results suggest that MSUC is a mechanism triggered during the early stages of prophase-I. Furthermore, it indicates that most spermatocytes carrying unsynapsed trivalents would proceed normally into diplotene.

### Unsynapsed chromatin recruits a second group of MSUC/MSCI-related proteins at early–mid pachytene

**Figure 4:**
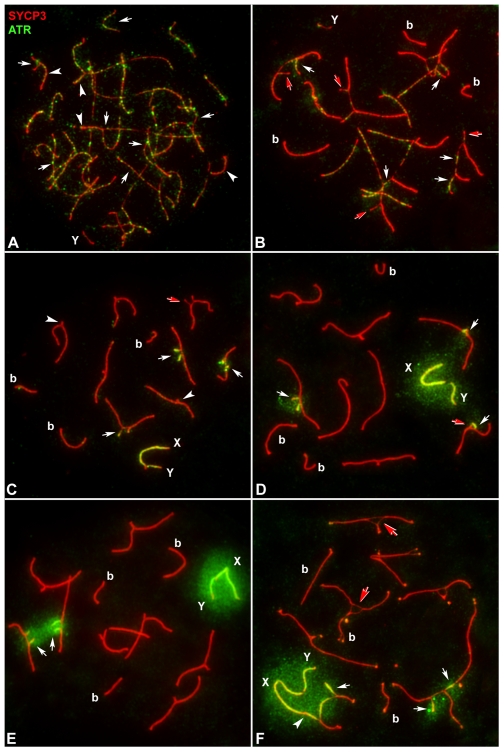
Localization of ATR (green) and SYCP3 (red) during prophase-I. (A) Early zygotene. ATR appears as foci localized in both the unsynapsed AEs (arrows) and the synapsed regions (arrowheads) of all chromosomes, excepting the Y chromosome. (B) Late zygotene. ATR foci are restricted to the AEs of unsynapsed chromosomes (white arrows). However, some unsynapsed AEs do not show ATR signal (red arrows). The Y chromosome (Y) shows a single ATR dot. (b) Bivalents. (C) Early pachytene. ATR appears as an irregular line running all along the unsynapsed AEs of open trivalents (white arrows) and the sex chromosomes (X, Y), although some open trivalents lack labeling (red arrows). Closed trivalents (arrowheads) do not show ATR labeling. (D) Mid-late pachytene. ATR localizes along the AEs of open trivalents (white arrows) and the sex chromosomes (X, Y) and becomes detectable in the surrounding chromatin. A trivalent shows a short unsynapsed AE without ATR signal (red arrow). (E) Late pachytene. ATR is detected along the unsynapsed AEs of trivalents and the sex chromosomes (X, Y) and is intensely distributed in the surrounding chromatin (arrows). (F) Early diplotene. Autosomes begin desynapsis. ATR localizes along the AEs and the surrounding chromatin of two trivalents (arrows) and the sex chromosomes (X, Y). One of the trivalents is ectopically associated with X chromosome (arrowhead). Newly desynapsed autosomes lack ATR labeling (red arrows).

Next, we investigated the presence of ATR in the unsynapsed regions of trivalents (Figure 4, Figure S2, and Figure S3). During zygotene, ATR labeling appeared as small foci located on the AEs/LEs in both synapsed and unsynapsed autosomes and in the X chromosome, but it was rarely observed in the Y chromosome (Figure 4A). At the zygotene/pachytene transition, ATR began to disappear from the chromosomes that had completed their synapsis (Figure 4B), although it remained as numerous and intense foci on the unsynapsed AEs. At this stage, a single ATR focus was always detected on the Y chromosome.

During early pachytene, ATR labeling appeared as a continuous line along the unsynapsed AEs of trivalents and sex chromosomes (Figure 4C). ATR localization contrasted with that of γH2AX, the latter including the whole unsynapsed chromatin (Figure 3C). This result indicates absence of colocalization of the two proteins during late zygotene and early pachytene in the unsynapsed regions (Figure S2). On the other hand, we observed some unsynapsed trivalent regions without the ATR signal (Figure 4B–4D). This observation is consistent with the absence of γH2AX in some unsynapsed trivalent regions and strongly suggests the existence of two classes of unpaired chromosome segments during early pachytene: one class that shows neither γH2AX nor ATR protein, and another class that shows the presence of both proteins.

At mid-pachytene, ATR labeling was still apparent along unsynapsed AEs and also appeared to extend to the surrounding chromatin of the unsynapsed regions of trivalents and of the sex chromosomes (Figure 4D); then, it became brighter as spermatocytes progressed to late pachytene (Figure 4E). During diplotene, ATR remained visible on the surrounding chromatin of the sex chromosomes and of the open trivalents (Figure 4F); its signal progressively faded and completely disappeared by the late diplotene stage.

**Figure 5:**
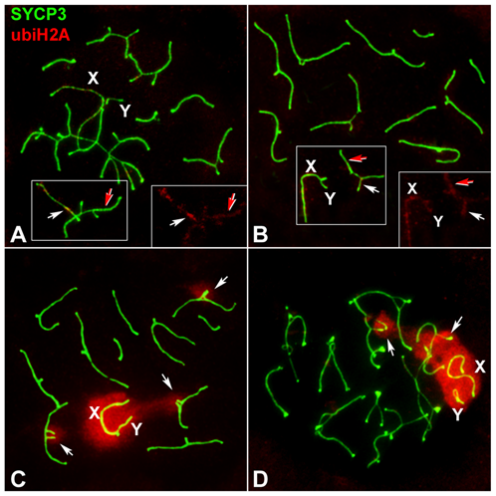
Localization of ubiH2A (red) and SYPC3 (green) during prophase-I. (A) Zygotene. UbiH2A is mostly absent, excepting for a weakly labeling localized along some synapsed LEs (red arrows) and unsynapsed AEs (white arrows). This labeling is only occasionally detected. Right insert represents the ubiH2A signal on the squared area. (B) Early pachytene. UbiH2A is absent although it may occasionally localize along the unsynapsed AEs of the autosomes (white arrows), the sex chromosomes (X, Y), and the synapsed LEs (red arrows). (C) Mid pachytene. UbiH2A intensely labels the chromatin of sex chromosomes (X, Y) and the chromatin surrounding unsynapsed AEs of trivalents (arrows) and is absent in the synapsed chromatin. (D) Diplotene. UbiH2A persists in the chromatin surrounding unsynapsed chromosomes (arrows) and the sex chromosomes (X, Y) whereas it is absent in the chromatin around desynapsed segments of homologous chromosomes.

**Figure 6:**
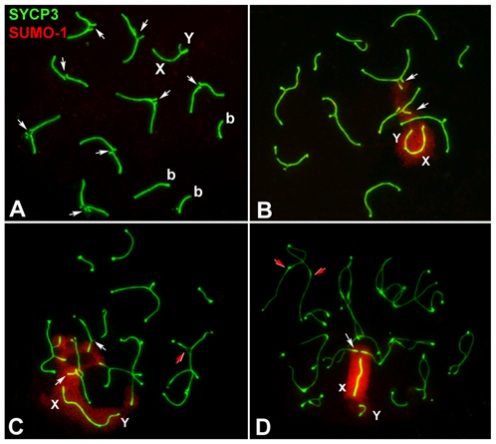
Localization of SUMO-1 (red) and SYCP3 (green) during prophase-I. (A) Early pachytene. SUMO-1 is not detected in both the chromatin of unsynapsed trivalents (arrows) and the XY body. (b) Bivalents. (B) Mid pachytene. SUMO-1 is only present in the chromatin of unsynapsed trivalents (white arrows) and the sex chromosomes (X, Y). (C) Early and (D) late diplotene. SUMO-1 persists in the unsynapsed chromatin (white arrows) and it is not present in the separated segments of homologous chromosomes (red arrows).

**Figure 7:**
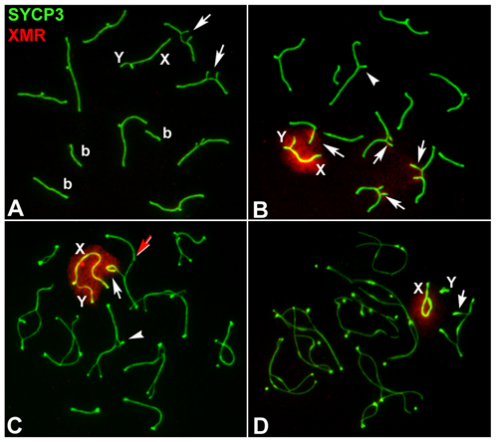
Localization of XMR (red) and SYCP3 (green) during prophase-I. (A) Early pachytene. XMR is not detected in both the chromatin of unsynapsed trivalents (arrows) and the XY body. (b) Bivalents. (B) Mid pachytene. XMR is present in the chromatin sex chromosomes (X, Y), while faint labeling is also detected in the unsynapsed regions of open trivalents (arrows). XMR labeling in the trivalents associated to the sex body is more intense than in those that are not associated. (C) Early diplotene. XMR persists in the unsynapsed chromatin of the trivalents (arrows) and the sex chromosomes and is not present in the segments that start desynapsis (arrowhead). Notice that a fully synapsed acrocentric in an open trivalent can elude the deposit of XMR (yellow arrow). (D) Late diplotene. XMR tends to disappear and is almost undetectable at the end of diplotene.

In view of these results, we next analyzed the pattern of appearance and localization of three other MSUC/MSCI-related proteins: 1) monoubiquitinated H2A histone (ubiH2A) (Figure 5), known to be associated with transcriptional silencing of unsynapsed autosomes and sex chromosomes in mouse male meiosis [18]; 2) SUMO-1 (Figure 6), which is involved in SC assembly as well as in the formation of the sex body [30], [66]–[68], and 3) XMR (Figure 7), a member of the *XLR* gene superfamily [69], known to localize in the sex body [27].

We found that during zygotene (Figure 5A) and early pachytene (Figure 5B), ubiH2A appeared as a faint signal on the chromosome ends on both synapsed and unsynapsed LEs, as previously observed [18]. On the contrary, at mid-pachytene, an intense labeling appeared on the chromatin of unsynapsed segments of trivalents and of the sex chromosomes (Figure 5C), persisting until mid diplotene (Figure 5D), when it started to disappear.

SUMO-1 was not detected during zygotene (data not shown) and very early pachytene spermatocytes (Figure 6A). It appeared on the unsynapsed chromatin of sex chromosomes and trivalents during a temporal window between the early to mid-pachytene transition (Figure 6B–6D), indicating that its appearance was delayed compared to mice with standard karyotype [30],[70], and it remained detectable until the end of diplotene.

XMR started to accumulate on the unsynapsed chromatin of trivalents and of the sex chromosomes at early to mid-pachytene transition and it disappeared at late diplotene (Figure 7). Interestingly, the intensity of the XMR signal seemed to be lower on the unsynapsed chromatin of the trivalents that were far from the sex chromosomes compared to that on the unsynapsed chromatin of the trivalents that were close to the sex body. These results suggest that the location of XMR in the open trivalents could be influenced by their association with the sex chromosomes.

**Figure 8:**
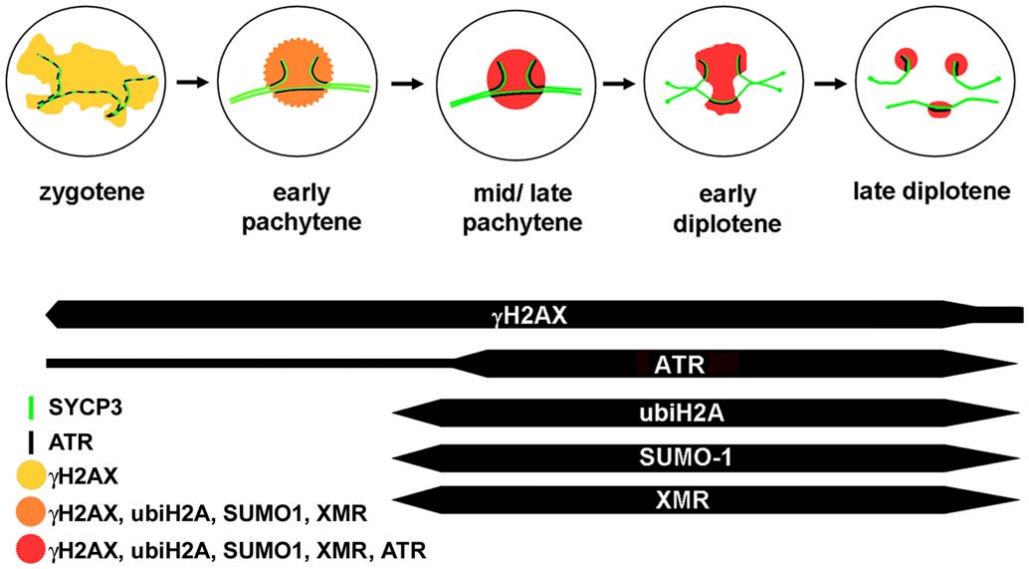
Dynamic recruitment of MSUC proteins in unsynapsed chromatin of Robertsonian trivalents. γH2AX appears at zygotene distributed in the whole chromatin while ATR appears as discrete foci scattered along the AEs/LEs as revealed by SYCP3 protein. At the beginning of pachytene, γH2AX becomes restricted to the chromatin surrounding unsynapsed AEs. Accumulation of SUMO 1, monoubiquitinated H2A, and XMR on unsynapsed chromatin occurs through the early pachytene stage, before the accumulation of ATR in the unsynapsed chromatin at mid-pachytene. These proteins remain associated to the unsynapsed chromatin until late diplotene, indicating that spermatocytes with unsynapsed trivalents skip pachytene arrest and progress further into later stages of meiosis. Newly desynapsed chromosomal regions at diplotene do not incorporate any of the MSUC-related proteins.

In summary, our results show that the proteins γH2AX and ATR started to appear at the beginning of prophase I (leptotene), but intense labeling of ubiH2A, SUMO-1 and XMR was detected at a later stage (early to mid-pachytene). The appearance of ubiH2A and SUMO-1, which are known to be involved in DNA repair [71],[72] slightly preceded the spread of ATR from the chromosome axes to the unpaired chromatin (see Figure S3 for a comparison between the timing of the appearance of SUMO-1 and ATR on unsynapsed chromatin). Therefore, ATR was the last protein to appear on the unsynapsed chromatin at mid-pachytene (Figure 8). All these proteins remained localized on the unsynapsed regions of trivalents and of the sex chromosomes until late diplotene, indicating an active repair of DNA on the unsynapsed chromatin of trivalents. Also, at mid-pachytene, ubiH2A and SUMO-1 might be involved in determining those chromatin modifications that would lead to the transcriptional inactivation of unsynapsed chromatin [72],[73].

### Spermatocytes with open trivalents predominate during the first meiotic prophase

The presence of trivalents with unsynapsed proximal regions throughout the first meiotic prophase raises the question of how many of these trivalents achieve a complete synapsis. To this end, we analyzed the number of completely synapsed (closed) and partially unsynapsed (open) trivalents during early, mid- and late pachytene and during early and mid-late diplotene (Table 1) in two individuals. No statistical differences were found between them. At the beginning of pachytene, only 2.22% of spermatocytes had completed synapsis in all trivalents, whereas most spermatocytes showed one to eight open trivalents, with spermatocytes having four open trivalents occurring at the highest frequency (27.41%). During prophase progression, the frequency of spermatocytes with a high number of open trivalents tended to decrease, even though, at late pachytene, the great majority (87.55%) of spermatocytes possessed open trivalents (Table 1). At diplotene, the frequency of spermatocytes with closed trivalents or with one open trivalent (recognized by the presence of γH2AX) increased slightly (18.36% and 51.20%, respectively), although not significantly when compared to that of late pachytene. On the contrary, the frequency of spermatocytes with two, three and four open trivalents slightly decreased (Table 1). These data show that most spermatocytes maintained partially unsynapsed trivalents throughout pachytene, although their number decreased towards the end of pachytene along with an increase of spermatocytes with completely synapsed trivalents.

### Spermatocyte elimination preferentially occurs at the metaphase, not at the pachytene stage

**Figure 9:**
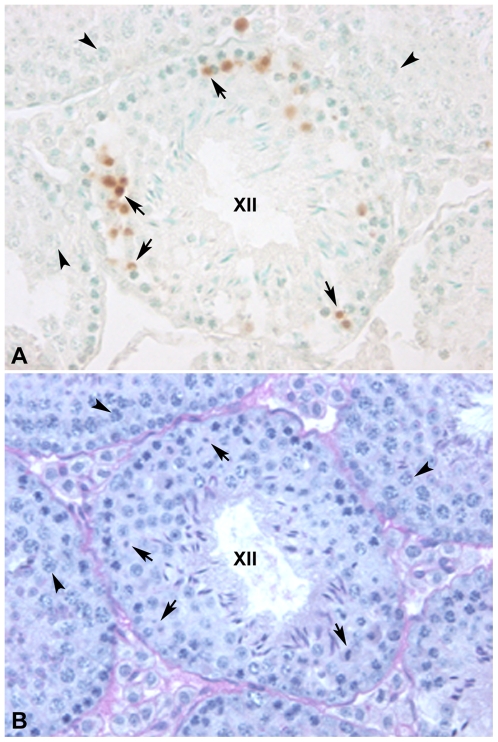
Cross-section of a stage XII tubule of the cycle of the seminiferous epithelium showing germ cell death of meiocytes in Robertsonian heterozygous mice. (A) Metaphases are the most frequent cell type positive to the TUNEL assay (arrows). Notice the absence of apoptotic cells in the surrounding tubules that are at different stages of seminiferous epithelium, and contain abundant pachytene spermatocytes (arrowheads). (B) Periodic-acid-Schiff (PAS) reaction and haematoxylin counterstaining of the same tubule cross-section.

To estimate germ cell death, we made a quantitative evaluation of the TUNEL-positive cells present in seminiferous tubule cross-sections. Confirming previous results [39],[49], TUNEL positive cells were almost exclusively present in the meiotic compartment of stage XII of the seminiferous epithelium, which contains spermatocytes at the zygotene-pachytene transition, metaphase I and II. An average of 19.44% (±4.37) of spermatocytes were TUNEL-positive, most of which were at the metaphase stage (Figure 9). When we specifically evaluated metaphase cells at stage XII, we found that 63% of them were TUNEL positive, as shown in an our previous study [39]. This suggests that 37% of metaphase cells are able to pass the spindle checkpoint and progress to further stages of differentiation. In this regard, we previously reported a mean ratio between round spermatids and pachytene spermatocytes of 1.43, corresponding to 36% of germ cell survival following meiosis in the same type of Rb heterozygous mice, although in the homozygous parentals germ cell survival is 84% and 86% for 2n = 40 and 2n = 24 karyotypes, respectively [40],[43]. Moreover, the absence of extensive cell death in other stages of the spermatogenetic cycle suggests that pachytene and diplotene spermatocytes are able to progress to meiotic divisions despite the presence of unsynapsed trivalents.

## Discussion

The aim of this study was to evaluate the involvement of MSUC during the meiotic progression of spermatocytes of Rb heterozygous mice. The data presented here show that the mechanisms that regulate MSUC are active during meiosis in mice heterozygous for multiple simple Rb translocations. We report that most pachytene spermatocytes bear trivalents with unsynapsed regions that incorporate, in a stage-dependent manner, proteins involved in MSUC. Our results demonstrate that although many chromosomal regions remain unsynapsed, massive cell death is not detected at pachytene. On the contrary, spermatocytes bearing unsynapsed chromosomes subject to MSUC progress into diplotene.

### Synapsis and recombination progression

It has been repeatedly reported that synapsis is delayed in heterozygotes for Rb translocations [44], [45], [47], [51]–[54],[57] and other chromosomal rearrangements [46],[74],[75]. The results presented here are in agreement with these previous reports. During zygotene, while synapsis progresses rapidly in the bivalents, in the trivalents it is initiated at the distal ends and then slowly progresses to the proximal ends of the acrocentrics. Previous reports have suggested that a delay in synapsis might be influenced by architectural constraints [43],[52],[58],[76]. In fact, the centromeres and proximal telomeres of acrocentric chromosomes are located at the nuclear periphery, while centromeres of metacentric chromosomes are located more internally in the nucleus (unpublished observations). This distinct localization of centromeres is defined by the different trajectory of the metacentric chromosomes' AEs within the nuclear space compared to that of acrocentrics' AEs [76],[77]. As synapsis of trivalents progresses from their distal telomeres, metacentric centromeres tend to approach to the nuclear envelope, where acrocentric centromeres and proximal telomeres are bound. These circumstances would explain why at the beginning of pachytene, while bivalents and sex chromosomes have achieved their respective synapsis, trivalents still appear with an open configuration. The presence of many unsynapsed proximal regions in acrocentric chromosomes located at the nuclear periphery would promote their association, causing the appearance of an ectopic heterologous synapsis between them or with the sex chromosomes at early pachytene. Most of these associations tend to disappear as trivalents complete their synapsis during mid and late pachytene. In agreement with previous reports [45],[52], our results show a decrease in the number of open trivalents throughout pachytene. These results suggest that trivalents can complete synapsis during the mid and late pachytene stages, as previously reported by Moses and coworkers [52]. However, contrary to the results found in lemur Rb heterozygotes [52], in which all trivalents finally achieve complete synapsis, in mouse there is a striking persistence of trivalents in open configuration throughout pachytene and at later stages [45].

Compared to autosomal bivalents, we found that trivalents retain RAD51 until later stages; however, RAD51 foci are not specifically enriched in the unsynapsed segments of the trivalents, a finding that differs from previous studies that have reported the maintenance of this protein on asynaptic autosomal segments [78],[79]. RAD51 finally disappears from the trivalents during mid-pachytene, despite the presence of unsynapsed segments. This circumstance has two interesting implications. First, our results confirm previous data that cells can accomplish prophase-I with unsynapsed autosomes [1],[33],[36],[64],[80],[81]. Since completion of the recombination/repair process is considered necessary to bypass the pachytene checkpoint [2],[4],[5],[82] it is likely that unsynapsed segments are repaired by the end of pachytene. This behavior parallels the situation found in the sex chromosomes. Second, some trivalents probably complete synapsis after RAD51 has disappeared, indicating the existence of a mechanism that is able to complete synapsis independently of the usual recombination/repair pathway [83]. Interestingly, many of these late synapsis events culminate with the heterologous synapsis of acrocentric chromosomes within each trivalent. This process, called synaptic adjustment, has been previously reported for these and other chromosomal rearrangements [44],[45],[52],[78],[84]. An additional consequence of both the persistence of unsynapsed and the presence of non-homologous synapsed chromosome regions is the reduction of chromosome segments where reciprocal homologous recombination could take place. This could account, at least partially, for the displacement of chiasma from the centromeric regions and the overall decrease of recombination frequency observed in Rb heterozygotes [57],[85].

### Dynamics of chromatin modifications involved in MSUC

The results presented here show that the unsynapsed regions of trivalents incorporate many of the proteins related to MSUC, such as γH2AX, ATR and ubiH2A [17],[18] and some markers that have been previously reported only in association with MSCI, such as SUMO-1 and XMR [27],[30], supporting the idea that MSCI could be a particular case of MSUC [17],[18].

Our study on Rb heterozygotes reveals further interesting features of the MSUC process. We found that during early pachytene, some unsynapsed regions do not exhibit either γH2AX or ATR signals. This labeling is especially striking in those trivalents in which one of the open acrocentrics incorporates these markers while the other does not (see Figure 3D). This absence of either γH2AX or ATR signals might be due to a limited availability in the meiocytes of factors triggering MSUC and MSCI, like BRCA1 and ATR, as recently suggested [33],[36]. However, alternative explanations could be formulated taking into account that: 1), unlabeled unsynased chromosome segments are found in cells with either a high or a low number of open trivalents; 2), we never observed MSCI to be hampered in the sex chromosomes. Since the absence of either γH2AX or ATR labeling on some unsynapsed regions is mainly found at early pachytene, we favor the interpretation that unlabeled chromatin could represent chromosomal regions that are about to synapse and/or are asynaptic but MSUC is not initiated yet. In our model, asynapsis could not be extensive enough to exhaust MSUC/MSCI triggering factors; asynapsis in each trivalent affects just a short chromosome length, thus the total amount of unsynapsed chromatin in Rb heterozygous mice is lower than in other mouse models [33],[36]. However, given the physiological interdependence of spermatocytes in the seminiferous epithelium provided by the presence of intercellular bridges [86]–[88], it is also likely that cytoplasmic flux could compensate the mRNA/protein levels of MSUC components among different cells, buffering the effect of extensive asynapsis in some spermatocytes. These facts could determine the success of spermatocytes to have a normal MSUC/MSCI performance during the first prophase and will serve to avoid stage IV pachytene apoptosis.

Our study also adds new clues to the understanding of the sequence of initiation and spreading of chromatin modifications involved in MSUC. H2AX phosphorylation detected at late zygotene was the first modification found in unsynapsed chromatin. This was followed by the accumulation of ubiH2A, SUMO-1, XMR and finally ATR on these regions during the early-mid pachytene transition. Thus, we suggest that the modifications of the chromatin involved in MSUC occur in at least two phases (Figure 8). The first phase initiates with the phosphorylation of H2AX, resulting in chromatin silencing at leptotene/zygotene. The second phase starts at early-mid pachytene with a second round of chromatin modifications, probably driven by the persistence of ATR at unsynapsed AEs, and it involves the incorporation of ubiH2A, SUMO-1, XMR, and finally ATR into unsynapsed chromatin. Whether it also involves other histone replacements and/or modifications, such as histone H3.1 and H3.2 replacement by H3.3 and H3, and H4 methylation [26], or the incorporation of other specific proteins or RNAs, remains to be determined.

Our analysis of the temporal appearance and localization of the proteins involved in MSUC has shown that ATR starts to spread over the chromatin of unsynapsed trivalents only at mid-pachytene, after the massive accumulation of γH2AX, while ubiH2A, SUMO-1, and XMR accumulate throughout early pachytene. Previous studies have suggested that ATR is involved in phosphorylating H2AX on the surrounding chromatin at late zygotene [17],[21] and that XMR and SUMO-1 accumulate on the sex body during early pachytene [27],[30],[70]. Although the pattern of appearance of some of these proteins is not completely established and discrepancies have been reported by different authors [17],[21],[30],[70],[89],[90], the comparison of these studies with our results suggests that: 1) the incorporation of many MSUC-related factors is delayed in Rb heterozygotes compared to homozygotes; and 2) our cytological approach, and previous studies [89],[91], are not completely congruent with the role of ATR in phosphorylating H2AX at late zygotene. Since we cannot rule out that undetectable amounts of ATR are present in the unsynapsed chromatin at late zygotene, other methodological approaches would be necessary to confirm this issue.

Finally, our results indicate that in mouse MSUC is triggered during zygotene-early pachytene and that desynapsing LEs at diplotene do not incorporate MSUC markers, even if they are adjacent to regions that have remained unsynapsed during pachytene. This differs from the recently reported dynamics of sex chromosome inactivation in chicken females, in which two waves of H2AX phosphorylation, one at zygotene and other one at late pachytene, have been detected [92]. These differences in MSUC dynamics open interesting questions in an evolutionary context.

### Spermatocytes with unsynapsed trivalents avoid pachytene arrest

Meiotic failure has been postulated as one of the main causes of infertility in organisms bearing chromosomal rearrangements. Several models have been proposed to explain this phenomenon, including the alteration of transcriptional activity of autosomes and sex chromosomes [1], [17], [34], [93]–[95], the impairment of synapsis and recombination progression [8],[42],[45],[75],[82],[96], the alteration of nuclear architecture during prophase-I [43], and the incorrect orientation and segregation of chromosomes during meiotic divisions [39],[49],[50].

Current models postulate the existence of a pachytene checkpoint that monitors synapsis and/or recombination progression [2],[4],[5]. Pachytene arrest resulting from asynapsis has been proposed to occur as a consequence of MSUC through the inactivation of genes that are crucial to meiotic progression [17]. Additionally, it has been suggested that sequestration MSUC-related proteins like BRCA1 and ATR resulting from an excess of asynaptic chromosomes might prevent their relocation to the sex chromosomes, hampering MSCI initiation in males [1],[33] and an extensive MSUC response in females [36]. The subsequent inability to inactivate the sex chromosomes has been proposed as a primary cause of spermatocyte apoptosis in a variety of mouse models [1],[33]. The presence of many open trivalents in our model does not result in sequestration of repair factors such as ATR on unsynapsed autosomal regions, allowing the correct progression of MSCI. These results indicate that in our model asynapsis *per se* could not be sufficient to trigger pachytene arrest. This agrees with recent reports on human [64],[80],[81] and mouse meiosis [1],[33],[36] indicating that cells can “tolerate” a limited degree of asynapsis. Therefore, it seems likely that there is not an stringent synapsis-specific checkpoint acting during pachytene in mouse and that MSUC involvement in triggering a checkpoint during prophase-I through MSCI hampering could be limited to extreme asynaptic situations.

Nevertheless, we consider important to stress that the impairment of the meiotic progression of spermatocytes with synaptic defects could still rely on the deregulation of gene expression caused by MSUC. In this sense, MSUC effects would greatly depend on the number and/or nature of genes that are transcriptionally inactivated [17]. In Rb heterozygotes, the unsynapsed segments comprise the pericentromeric heterochromatin-rich regions and euchromatic regions meager in genes, most of which might not be critical for meiosis progression and subsequent spermiogenesis. However, while MSUC has little effect in determining pachytene arrest in this model, it is likely that the effect could be much more relevant in other models.

### Spermatocyte death is mainly observed during meiotic divisions

We found that in Rb heterozygotes meiotic failure occurs mainly during meiotic divisions, as we recorded a high proportion of apoptotic cells at stage XII of the seminiferous epithelium and very few TUNEL-positive pachytene spermatocytes. We are aware that apoptotic pachytene cells are very rapidly removed and difficult to document by TUNEL [50]. On the other hand, metaphase apoptotic cells may be difficult to eliminate from the seminiferous epithelium, causing and overestimation of cell dead at these stages [47]. However, our result are in agreement with previous reports showing that in Rb heterozygotes bearing trivalents or complex rings cell death is mainly found during meiotic divisions [47]–[49] while cell death mainly occurs during prophase-I in Rb heterozygotes bearing chromosome chains [41], [46]–[48]. Furthermore, the absence of massive cell death at the pachytene stage is also supported by our previous studies [39],[40], which showed only a slight reduction of the number of this type of spermatocytes from stage I to XI of the cycle of the seminiferous epithelium. This could account for the elimination of those spermatocytes with a high number of open trivalents, whereas those that have one to four open trivalents might be able to bypass pachytene arrest and proceed to further stages. Therefore, meiotic failure in our Rb heterozygotes seems to rely mainly on the action of checkpoints during metaphase I and II [39],[40],[45],[49]. Trivalents may have difficulties in achieving a correct orientation on the meiotic spindle, determining a delay of anaphase initiation that would lead to cell degeneration [7],[49],[97] and subsequent reduction of fertility.

### Evolutionary perspectives

Paradoxically, despite the reduced fertility of heterozygous mice, Rb translocations are very frequent in wild populations [41],[98], spread rapidly [99],[100] and represent one of the main causes of karyotype evolution in mammals [101]. We propose that the circumvention of pachytene arrest even in the presence of chromosome regions subjected to MSUC, as demonstrated in the present study, could contribute to increasing the chances of many spermatocytes to reach meiotic divisions and to differentiate into viable sperm. Although substantial cell death is produced at the metaphase stage (up to 63%), the chances of producing viable gametes are still much higher than if a more stringent pachytene checkpoint were able to eliminate up to 87% (Table 1) of pachytene spermatocytes bearing unsynapsed chromosomes.

In an evolutionary context, it must be stressed that when a chromosomal rearrangement arises in a natural population, the rearranged chromosomes must still pair, synapse, recombine and segregate from their cognate homologues. Therefore, the possibility that a chromosomal rearrangement will spread into a population would greatly depend on the meiotic defects it may cause in the heterozygotes. Thus, while Rb rearrangements may have a relatively mild effect on mouse pachytene progression, for other chromosomal rearrangements and organisms, this model cannot be applied [102].

## Materials and Methods

### Mice

Heterozygous Robertsonian mice (*2n = 32*, eight Robertsonian chromosomes in a heterozygous state) were generated by mating females of the laboratory strain CD1 (*2n = 40*, all acrocentric chromosomes) and males of the Milano II race (2n = 24, eight pairs of Robertsonian metacentrics in a homozygous state, Rb (2.12), Rb (3.4), Rb (5.15), Rb (6.7), Rb (8.11), Rb (9.14), Rb (10.13), Rb (16.17). Six three-month old male mice were analyzed. Mice were maintained at 22°C with a light/dark cycle of 12/12 hours and fed *ad libitum*. Procedures involving the use of the mice were approved by the animal ethics committees of the Faculty of Medicine, University of Chile, and the University of Pavia (Italy).

### Immunofluorescence

Spermatocyte spreads and squashes were obtained following the procedures described by Peters et al. [103] and Page et al. [104]. The slides were placed in PBS and incubated with the following primary antibodies: mouse anti-SYCP3 1∶100 (Abcam, Ab12452); rabbit anti-SYCP3 1∶100 (Abcam, Ab15093); rabbit anti-SYCP1 1∶100 (Abcam, Ab15087); rabbit anti RAD51 1∶50 (Calbiochem, PC130); mouse anti-phospho-histone H2AX (Ser139) 1∶1000, clone JBW301 (Upstate, 05–636); goat anti ATR 1∶80 (Santa Cruz Biotechnology, sc-1887); mouse anti ubiquityl-histone H2A 1∶15, clone E6C5 (Upstate, 05–678); mouse anti GMP-1 (SUMO-1) 1∶50 (Zymed, 33–2400); mouse RIK2D3 1∶100 that recognizes the XMR protein in the testis [27], kindly provided by Denise Escalier (Université Paris 5, Paris, France). After rinsing in PBS, the slides were incubated with appropriate secondary antibodies diluted 1∶100 in PBS: FITC-conjugated donkey anti-rabbit IgG, FITC-conjugated donkey anti-mouse IgG, TR-conjugated donkey anti-mouse IgG and FITC-conjugated donkey anti-goat IgG. Slides where then stained with 1 µg/ml DAPI. After a final rinse in PBS, the slides were mounted with Vectashield. Observations were made in a Nikon (Tokyo, Japan) Optiphot or an Olympus BX61 microscope equipped with epifluorescence optics and the images were photographed on DS camera control unit DS-L1 Nikon or captured with an Olympus DP70 digital camera. All images were processed with Adobe Photoshop CS software.

### Three-dimensional reconstruction of squashed spermatocytes

Immunolabeled spermatocytes were observed in an Olympus BX61 microscope equipped with a motorized Z-axis, epifluorescence and an Olympus DP70 digital camera. A collection of optical sections were captured using the analiSYS software (Soft Imaging System, Olympus). Images were subsequently analyzed and processed using the public domain software ImageJ (National Institutes of Health, United States; http://rsb.info.nih.gov/ij), and the output video files were edited with VirtualDub (VirtualDub, http://www.virtualdub.com).

### Histology and TUNEL assay

The right testis of three mice were fixed in Bouin's fluid and embedded in paraffin wax. Five-micrometer serial transverse cross-sections were made and at least four serial sections *per* testis were mounted on each glass slide. One slide was stained by the periodic-acid-Schiff (PAS) reaction and counterstained with haematoxylin to identify the stages of seminiferous epithelium according to Oakberg [105]; the other slide was processed with the terminal deoxynucleotidyl transferase-mediated dUTP nick end-labelling (TUNEL) method, using an ApopTag Plus Peroxidase In Situ Apoptosis Kit (Chemicon-Millipore, Billerica, USA), according to the manufacturer's instructions. Positive and negative controls were also set up. The positive controls were established using the slides contained in the same kit and following the manufacturer's instructions. For the negative controls, sections were processed without TdT enzyme in the labelling reaction mix. The sections were counterstained with 0.5% (w/v) methyl green for 10 min at room temperature. For each animal testis, 100 cross-sectioned tubules were scored to evaluate the frequency of apoptotic tubules. A cross-section of a tubule was considered apoptotic when three or more TUNEL-positive spermatocytes were present within the seminiferous epithelium [39],[49]. The percentage of TUNEL positive cells was calculated taking into account the total number of spermatocytes per tubule section. Abercrombie's correction was applied to all cell counts [106].

### Quantitative and statistical analysis

We analyzed 724 and 415 spermatocytes from two three month-old heterozygous Robertsonian mice. The synapsed condition of heterologous region of Robertsonian trivalents was determined by morphological analysis identifying chromosomes with SYCP3 and the presence or absence of γH2AX positive signal in the chromatin. The data obtained from each mouse in each prophase I stage were summarized. Statistical significance between mice was assessed by the one way analysis of variance (ANOVA), followed by Tuckey post test. A Z test for two proportions was used to compare the number of spermatocytes between late pachytene, early diplotene and middle/late diplotene. In both statistical analyses a p value<0.05 was considered statistically significant with a confidence interval of 95%.

## Supporting Information

Figure S1 Localization of SYCP3 (green) and γH2AX (red) in an early pachytene spermatocyte. γH2AX labeling covers the chromatin of the unsynapsed trivalent regions, as well as the entire X chromosome, which is associated with two trivalents, one at the proximal end (arrow) and another at the distal end, where the pseudoautosomal region (PAR) is located. The Y chromosome, which appears self-synapsed, also presents an intense γH2AX labeling. An autosome presumably presents a break that appears labeled by γH2AX (arrowhead). The inset on the top left represents the putative synaptic relationships between the X chromosome and the trilavents, the self-synapsis of the Y chromosome and the extension of γH2AX labeling (in blue). The position of the centromeres has been inferred from DAPI staining of the chromatin (not shown).
(1.60 MB TIF)

Figure S2 Localization of SYCP3 (blue), ATR (green), and γH2AX (red) during prophase-I. (A–C) Early pachytene. ATR appears as an irregular line along the unsynapsed AEs of open trivalents (arrows) and the sex chromosomes (X, Y), although some open trivalents lack labeling (arrowheads). Closed trivalents (arrowheads) do not show ATR labeling. γH2AX intensely labels the chromatin surrounding those unsynapsed AEs labeled with ATR. (D–F) Mid pachytene. ATR localizes along the AEs of open trivalents (arrows) and the sex chromosomes (X, Y) and becomes detectable in the surrounding chromatin of these regions. γH2AX labeling still comprises a wider chromatin area than that of ATR. (G–I) Mid pachytene. ATR labeling becomes more intense on the chromatin surrounding unsynapsed AEs (arrows), and this labeling is almost coincident with that of γH2AX. (J–L) Late pachytene. ATR labeling is very intense in the unsynapsed chromatin. ATR and γH2AX labeling is completely coincident on the chromatin surrounding unsynapsed AEs (arrows).
(4.93 MB TIF)

Figure S3 Localization of SYCP3 (blue), ATR (green), and SUMO-1 (red) during prophase-I. (A–C) Early-mid pachytene. ATR appears as an irregular line along the unsynapsed AEs of open trivalents (arrows) and the sex chromosomes (X, Y), although some open trivalents lack labeling (arrowheads). Closed trivalents (arrowheads) do not show ATR labeling. SUMO-1 weakly labels the chromatin surrounding those unsynapsed AEs labeled with ATR. (D–F) Mid pachytene. ATR localizes along the AEs of open trivalents (arrows) and the sex chromosomes (X, Y) and becomes detectable in the surrounding chromatin. SUMO-1 labeling becomes more intense, and it still comprises a wide chromatin area than that of ATR. (G–I) Mid pachytene. ATR labeling becomes more intense on the chromatin surrounding unsynapsed AEs (arrows), and this labeling is coincident with that of SUMO-1. (J–L) Late pachytene. ATR labeling is very intense in the unsynapsed chromatin. ATR and SUMO-1 labeling is completely coincident on the chromatin surrounding unsynapsed AEs (arrows).
(3.99 MB TIF)

Video S1 Three-dimensional reconstruction of squashed spermatocytes at prophase-I labeled with SYCP3 (green), SYCP1 (red), and γH2AX (blue). At leptotene γH2AX distributes in the whole nucleus, whereas at pachytene and diplotene it accumulates on the sex chromosomes (XY) and on open regions of trivalents (arrows), which in turn are devoid of SYCP1 labeling.
(1.74 MB MOV)

Video S2 Three-dimensional reconstruction of a squashed pachytene spermatocyte labeled with SYCP3 (green) and γH2AX (red). γH2AX labeling appears on the open trivalents regardless whether they are associated (red arrows) or not (yellow arrow) to the sex chromosomes (XY). Closed trivalents are devoid of γH2AX labeling (white arrow). The trajectory of three trivalents and the sex chromosomes is represented in the right panel.
(0.49 MB MOV)
