# Supplementary figures and images for: A High Incidence of Meiotic Silencing of Unsynapsed Chromatin Is Not Associated with Substantial Pachytene Loss in Heterozygous Male Mice Carrying Multiple Simple Robertsonian Translocations
Source: PLoS Genet. 2009 Aug 28;5(8):e1000625. doi: 10.1371/journal.pgen.1000625 (PMC2726437; doi:10.1371/journal.pgen.1000625)

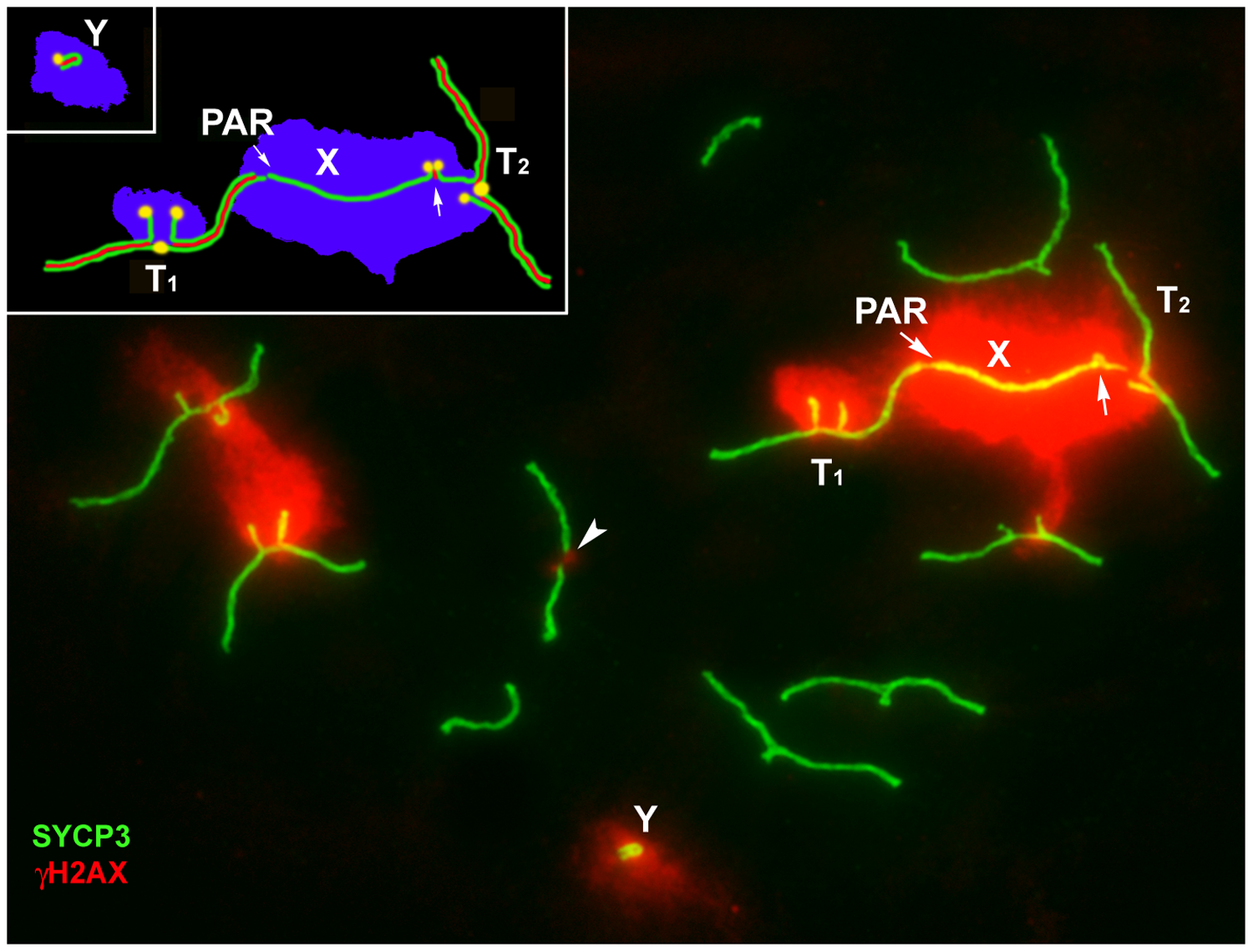

Supplement: Figure S1 — Localization of SYCP3 (green) and γH2AX (red) in an early pachytene spermatocyte. γH2AX labeling covers the chromatin of the unsynapsed trivalent regions, as well as the entire X chromosome, which is associated with two trivalents, one at the proximal end (arrow) and another at the distal end, where the pseudoautosomal region (PAR) is located. The Y chromosome, which appears self-synapsed, also presents an intense γH2AX labeling. An autosome presumably presents a break that appears labeled by γH2AX (arrowhead). The inset on the top left represents the putative synaptic relationships between the X chromosome and the trilavents, the self-synapsis of the Y chromosome and the extension of γH2AX labeling (in blue). The position of the centromeres has been inferred from DAPI staining of the chromatin (not shown). (1.60 MB TIF) [file pgen.1000625.s001.tif]

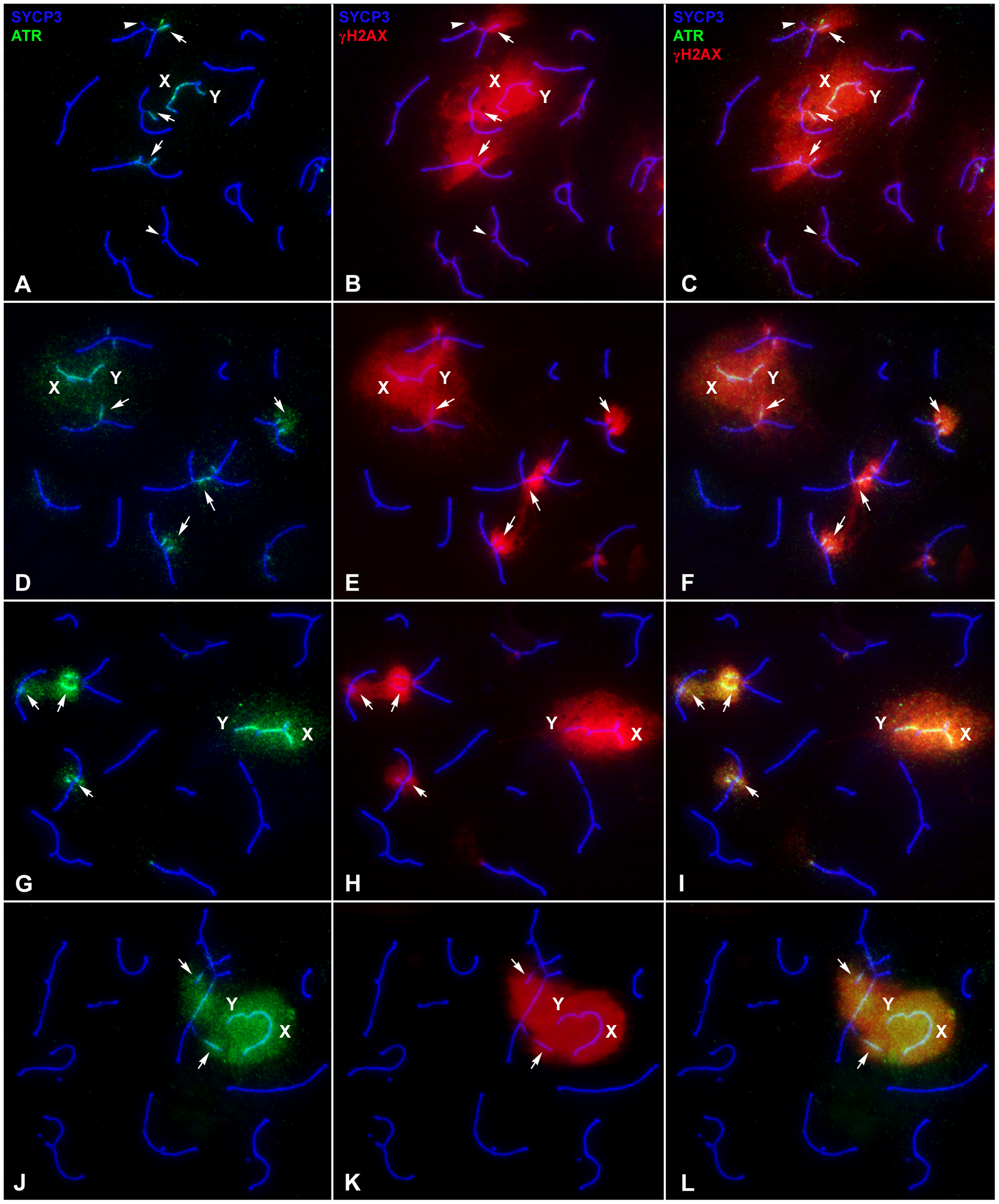

Supplement: Figure S2 — Localization of SYCP3 (blue), ATR (green), and γH2AX (red) during prophase-I. (A–C) Early pachytene. ATR appears as an irregular line along the unsynapsed AEs of open trivalents (arrows) and the sex chromosomes (X, Y), although some open trivalents lack labeling (arrowheads). Closed trivalents (arrowheads) do not show ATR labeling. γH2AX intensely labels the chromatin surrounding those unsynapsed AEs labeled with ATR. (D–F) Mid pachytene. ATR localizes along the AEs of open trivalents (arrows) and the sex chromosomes (X, Y) and becomes detectable in the surrounding chromatin of these regions. γH2AX labeling still comprises a wider chromatin area than that of ATR. (G–I) Mid pachytene. ATR labeling becomes more intense on the chromatin surrounding unsynapsed AEs (arrows), and this labeling is almost coincident with that of γH2AX. (J–L) Late pachytene. ATR labeling is very intense in the unsynapsed chromatin. ATR and γH2AX labeling is completely coincident on the chromatin surrounding unsynapsed AEs (arrows). (4.93 MB TIF) [file pgen.1000625.s002.tif]

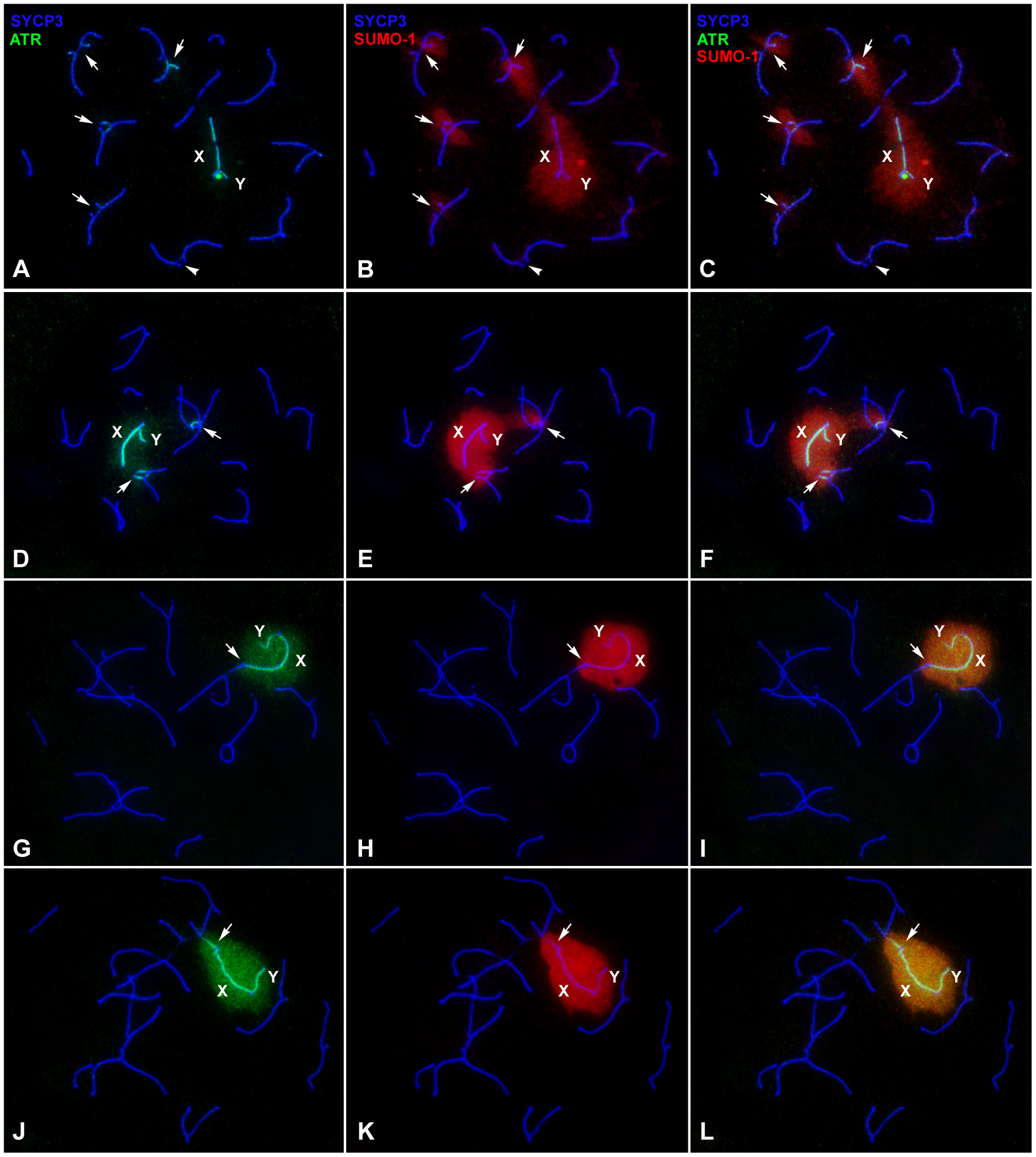

Supplement: Figure S3 — Localization of SYCP3 (blue), ATR (green), and SUMO-1 (red) during prophase-I. (A–C) Early-mid pachytene. ATR appears as an irregular line along the unsynapsed AEs of open trivalents (arrows) and the sex chromosomes (X, Y), although some open trivalents lack labeling (arrowheads). Closed trivalents (arrowheads) do not show ATR labeling. SUMO-1 weakly labels the chromatin surrounding those unsynapsed AEs labeled with ATR. (D–F) Mid pachytene. ATR localizes along the AEs of open trivalents (arrows) and the sex chromosomes (X, Y) and becomes detectable in the surrounding chromatin. SUMO-1 labeling becomes more intense, and it still comprises a wide chromatin area than that of ATR. (G–I) Mid pachytene. ATR labeling becomes more intense on the chromatin surrounding unsynapsed AEs (arrows), and this labeling is coincident with that of SUMO-1. (J–L) Late pachytene. ATR labeling is very intense in the unsynapsed chromatin. ATR and SUMO-1 labeling is completely coincident on the chromatin surrounding unsynapsed AEs (arrows). (3.99 MB TIF) [file pgen.1000625.s003.tif]
